# Horticultural therapy, nutrition and post-traumatic stress disorder in post-military veterans: developing non-pharmaceutical interventions to complement existing therapeutic approaches

**DOI:** 10.12688/f1000research.70643.1

**Published:** 2021-09-03

**Authors:** Richard Mottershead, Marjorie Ghisoni

**Affiliations:** 1 Ras Al Khaimah College of Nursing, RAK Medical and Health Sciences University, Ras Al Khaimah, P.O.Box 11172, United Arab Emirates; 2 School of Medical and Health Sciences, Bangor University, Bangor, Wales, LL57 2DG, UK

**Keywords:** Post-Traumatic Stress Disorder, Veteran’s Mental Health, Horticultural Therapy, Nutrition, Psychosocial Interventions.

## Abstract

Non-pharmaceutical interventions for veterans living with post-traumatic stress disorder are becoming a more popular way to address some of the social and personal needs identified by this group. Horticultural therapy or growing and eating food together provides several ways to increase mood, improve nutritional status, reduce loneliness and reduce the physical health impacts of mental illnesses such as post-traumatic stress disorder. In this paper we will discuss some of the issues people living with post-traumatic stress disorder might face. We will also provide an overview of the therapeutic effects of these approaches and how they will be applied in a locally identified group.

## Introduction

At present, in the United Kingdom (UK), there are former service-personnel leaving the military with no physical injuries but with underlying mental health problems. These may surface on return to the family unit, or at times of stress triggered by alcohol or homelessness. It was highlighted in a recent PhD thesis by one of the authors ( Mottershead, 2019) that the post military veterans interviewed feared for their sanity, suffered flashbacks, night terrors and experienced struggles with employment and families, reporting concerns for their partners or family. This is supported by Hossain *et al.* (2020) who in a review of the literature following a major crisis or trauma, revealed that there is a higher prevalence of mental health problems including anxiety, depression, substance misuse, suicidal ideation and post-traumatic stress disorder (PTSD) within the local population.

This is not a new concept for post military veterans, as similar problems have been reported as a result of the nations’ involvement in armed conflict since the commencement of the 21 ^st^ century. Harold Bridger (1985) suggested that the concept of non-pharmaceutical interventions arose in the autumn of 1942 at Northfield military Hospital in Birmingham, where a move to improve the return of neurotic casualties to military duty was tried out as a new form of treatment. The therapeutic community only lasted six weeks due to the chaos it created, but in that very short time it had given a glimpse of the success that could be achieved, and had been achieved previously in the Peckham experiment in the 1930s, with the creation of an” unintentional therapeutic community” ( Bridger, 1985. p.8.). In the following review of the literature, we will discuss how the non-pharmacological interventions of horticultural and nutrition therapy, can minimise, or reduce the physical and mental health effects of PTSD.

## Discussion

### Post-traumatic stress disorder

Military personnel exposed to war-zone stress are at significant risk of developing PTSD. Post-traumatic stress disorder (PTSD) has been studied in military personnel for more than 30 years, and PTSD may develop after an individual experiences or witnesses to a traumatic event, such as natural disasters or violent personal assault, life-threatening events such as terrorist attacks, violent crime and abuse, military combat, serious accidents or extended combat, exposure to certain environmental toxin ( Iribarren *et al*., 2005).

In 2015, a joint report by Help for Heroes and Kings Centre for Military Health Research (KCMHR), suggested that at least 10% of veterans who served over the last 20 years present mental health conditions that need treatment. The same report estimated that at least 61,300 out of 601,000 veterans who served as Regulars in the UK Armed Forces between 1991 and 2014 might suffer from mental health problems, and that this would require professional intervention (Help for Heroes & KCMHR, 2015). Amongst these mental health problems was post-traumatic stress disorder (PTSD). This is a psychiatric disorder that results from the experience or witnessing of traumatic or life-threatening events which thereby impairs the person’s daily life and can be life threatening ( Reisman, 2016). Moreover, Reisman (2016) explains that PTSD can cause significant disruption and interfere with personal and social performance, which subsequently leads to social withdrawal, anger, and aggression. Fear *et al.* (2010) demonstrated that PTSD in military personnel has a broad impact on military preparedness, and achievement of military objectives. However, data that identified a significant increase in PTSD symptoms at 3-year and 6-year follow up reviews on UK military veterans may be more worrying. Sareen (2014) resonates with this concern in highlighting scientific evidence and empirical research that suggests that military personnel with PTSD are at greater risk for more physical health problems, poorer health status, and higher medical service usage. Hence, much more research is needed on this perspective. Depression is the most common comorbidity of PTSD among military personnel ( Kessler *et al.*, 1995). A meta-analysis of 57 studies, conducted among both military and civilian samples, found a major depressive disorder and PTSD comorbidity rate of 52% ( Rytwinski *et al.*, 2013). Other prevalent psychiatric comorbidities of PTSD exhibited among military veterans include anxiety and substance abuse or dependence ( Hoge *et al.*, 2006).

Researchers have proposed several theories to explain the development of PTSD, including psychological theories, cognitive theories, emotional processing theory. Pan *et al.* (2018), suggests that the contemporary PTSD research aims to better understand the disease-related risk factor. They go on to suggest that there is convincing evidence of the relationship between abnormal levels of catecholamines, or stress hormones, in people living with PTSD, including the hormone norepinephrine as a result of facing fear and stress.

Furthermore, studies of risk factors of PTSD show that not everyone who experiences a traumatic event will develop PTSD, and therefore that there is role of individual factors in contributing to the development of PTSD ( Reisman, 2016). They also suggest that associated and identified individual and social risk factors include young age at the time of trauma, low socio-economic status and lack of support. More recently in a review by Herringa (2017) it was found that brain development in children can be affected by trauma in early life which increase the significance of psychological support and good nutrition for brain health in adulthood. The use of complementary and alternative medicine (CAM), such as horticultural therapy or nutrition advice, has been found to be on the increase in Sweden, as a way of developing coping skills in managing personal mental health needs ( Wemrell *et al.*, 2020). The management of nutrition and its effects on mental health is therefore a simple and easy way, alongside other complementary interventions, to improve resilience within the mind and body, and reduce the effects of living with long-term stress.

### Horticultural therapy

While horticultural therapy is not considered a first-line treatment for people living with PTSD, there are many studies that demonstrate improvement in overall health in the wider literature ( Detweiler *et al.*, 2015). In a pilot study on physical health and PTSD in veterans, Hall *et al.* (2020) found that being more active could improve the health outcomes for people living with PTSD, by reducing obesity and improving their mood. Similarly, Detweiler *et al.* (2015) found that horticultural therapy is an active, non-pharmaceutical intervention that can help people to improve their mood, reduce obesity and increase social interactions, often caused by isolation in veterans. Furthermore, they found that there was a notable improvement in scores on outcome measures such as quality of life and alcohol craving questionnaires. They go on to suggest that horticultural therapy as an extension of occupational therapy, has been found to have beneficial effects on both physical and mental health recovery. More recently, Wemrell *et al.* (2020) found in a review of complementary and alternative medicine (CAM) for people with mental illness in Sweden, that 14% of respondents were using horticultural therapy as an addition to their prescribed treatments.

The evidence above supports the findings of Cipriani *et al.* (2017), within their systematic review of the effects of horticultural therapy on persons with mental health conditions. This research revealed that horticulture can provide physical, neurological, and psychological rehabilitation, whilst improving motor skills as well as strength and endurance. They go on to suggest that being outside in the natural environment has been found to reduced stress and improve concentration and cognitive function. This has been known for some time in helping military personnel to return to civilian life. Wang (2010 p.4) cites Franklin Roosevelt in 1944, who in writing to the then secretary of war argued that, 

“ *No overseas casualty should be discharged from the armed forces until he has received the maximum benefit of hospitalisation and convalescent facilities, which must include physical and psychological rehabilitation, vocational guidance, pre-vocational training and re-socialisation”.*

One of the results of this was the application of a rehabilitation model by Dr Howard Rusk (cited in Blum & Fee, 2008), that was so successful that it was adopted by the military. Patients were not allowed to stay in bed, instead they were urged to get up and begin a re-conditioning program to develop residual capabilities. The flaw in the system was that while the goal was to make possible the integration of personnel with disabilities into society, the commitment ended at the hospital door. Once the doctors and therapists had done all they could, patients and families were on their own, and had to come to terms with society individually. Reisman (2016) argues that as the number of people with PTSD increases, there is still a need to develop a range of therapeutic approaches to help people living with PTSD, including complementary alternative medicine (CAM) in the community.

### Nutrition and brain health

In recent years, there has been more research on how the microbiome, or gut health, influence overall health, including the brain. Wang *et al.* (2018), in a review of the literature on systemic inflammation and neuroinflammation, found that consuming foods that contain phytochemicals that can cross the blood brain barrier (BBB), has been shown to reduce oxidative damage in the brain. Similarly, Bandyopadhyay (2021), in a review of neurodegeneration in the brain, found that there are certain influences upon brain health that can improve neurofunction by improving the function of the BBB and associated pathways. Poor brain health due to poor nutrition can cause damage to nerve cells within the body and the central nervous system (CNS), and to the part of the brain that can produce more nerve cells, the hippocampus ( Firth *et al.*, 2020). This can lead to negative effects on memory, mood and overall cognitive functioning. Kerage *et al.* (2019) in a review of the literature on the interaction between neurotransmitters and lymphocytes, found that there is direct communication between the two in response to threat, demonstrating that the brain can influence, and can be influenced, by the immune system. An overactive immune system together with overactive or damaged nerve cells can therefore lead to long term inflammation within the brain. People who experience chronic stress such as PTSD, may experience these symptoms as a sign of poor brain health ( McCarty & DiNicolantonio, 2016). This is caused by the brain becoming overexcited in response to stress hormones being produced in response to fear, and the oxidative stress damage caused by free radicals on the nerve cells ( Wang *et al.*, 2018). This is a natural response to stress via the vagus nerve which is part of the CNS, and the main communication channel between the brain and the other major organs in the body ( LaChance & Ramsey, 2018). An overactive stress response, as can be experienced by people with PTSD can therefore lead to widespread inflammatory damage around the body and within the brain ( Parker *et al.*, 2020). Good brain health is therefore highly dependent upon good nutrition and the absorption of nutrients in the body that can cross the BBB, together with a reduction of exposure to stressful environments. Growing and eating nutritious food can therefore aid in developing awareness of the importance of good nutrition for both physical and mental health ( Tanagra *et al.*, 2013).

### Gut-brain health

A whole food diet that is rich in nutrients can improve mental health and wellbeing in many ways, including improving brain health, improving digestive health, increasing the absorption and transport of nutrients around the body and eliminating toxins quickly and effectively ( Solovyev *et al.*, 2021). Whole foods include food that has been grown naturally and retains their beneficial phytochemicals and nutrients, which are either not present or removed in processed or refined foods. LaChance & Ramsey (2018), in a systematic review of the literature on a nutrient profiling system for depression, found that people who were deficient in Omega 3, Zinc, Magnesium, B vitamins, Vitamin C and A, and Selenium and Potassium were more likely to experience mental health problems. Horticultural therapy offers the opportunity to grow fresh, nutrient-rich food, and to control the amount of chemicals added, while at the same time develop individual knowledge about nutrition ( Tanagra *et al.*, 2013). In addition, fresh, home-grown food will contain more nutrients than food that has spent a long time in transportation. The gut is where the human body processes the food it ingests, an activity which is dependent upon millions of “friendly” bacteria ( Firth *et al.*, 2020). Feeding the friendly bacteria in the gut with whole food will also enable it to function better, and filter out toxins or absorb more nutrients, which in turn can improve mood and brain health. Solovyev *et al.* (2021) found in a review of the effectiveness of neural barriers, that the gut microbiome is important in breaking down nutrients for transportation to the brain and to prevent toxins reaching the BBB, where they can cause further damage. The also brain requires a lot of energy and hydration from food to function properly, so it is dependent upon a healthy gut that can process food efficiently and transport nutrients to the brain ( Bandyopadhyay, 2021). Most plant-based foods contain phytonutrients that are needed for a healthy nervous system and to remove toxins from the body ( Firth *et al.*, 2020). The brain also causes oxidative stress as it metabolises nutrients in nerve cells, which can cause inflammation if not removed quickly ( Li *et al.*, 2019). Anti-inflammatory and antioxidant foods that can be grown locally such as vegetables and fruit, are therefore essential for good brain health and can improve the functioning of the brain. The gut and the brain also produce the neurotransmitter serotonin, which in turn improves mood ( Bandyopadhyay, 2021). This synergetic process of digestion, cell production and repair and resilience, is essential all around the body including the gut and the brain ( Lin *et al.*, 2019). As a consequence of improving nutrient intake, the body will be able to fight off infection, reduce inflammation, improve mood and functions of all the major organs in the body, including the brain. Horticultural therapy alongside nutrition education can therefore become a very effective, non-pharmaceutical psychosocial intervention for people living with PTSD.

### Future considerations

This overview of the literature will inform the development of opportunities to take part in horticultural therapy with nutrition advice, for the well-being and rehabilitation of military veterans, reservists, emergency service personnel and their families in North Wales, UK ( Firth *et al.*, 2020). Instead of working in confined environments, the participants will be invited to join a small farm, Ty Gwalia, in the open countryside with gardens and greenhouses, and will aid in improving the re-integration and development of relationships through the execution of horticultural tasks, cooking together and other outdoor activities ( Detweiler *et al.*, 2015; Reisman, 2016). For those veterans making the transition to a civilian identity, the combination of directed physical work and the need to work as a team with others, provide a route back into civilian life and improve confidence, self-esteem and personalised wellbeing ( Hall *et al.*, 2020; Tanagra *et al.*, 2013). In addition, providing nutrition advice will also improve outcomes in physical and mental wellbeing, and help veterans to reduce the effects of stress and physical health problems ( Detweiler *et al.*, 2015). 

The Ty Gwalia project would aim to look at the problems encountered by people living with PTSD who have passed through the “hospital door”, and how a horticultural or land-based setting alleviates the problems encountered through involvement with new environmental and social situations ( Wemrell *et al.*, 2020). The project will be carried out by the registered UK charities Woody’s Lodge and Wintergreen UK - CIC. Both organisations offer complementary interventions, free of charge to local people with lived experience of PTSD including emergency workers, to support their recovery, and provide local mental health services. This has been found to have beneficial effects for people who want to personalise their recovery without needing further access to increased medical or other interventions ( Cipriani *et al.*, 2017).

## Conclusions

This review has highlighted an awareness of the benefits of horticultural and nutritional therapy, as a therapeutic treatment for mental health and mental disorders. However, in traditional health practice, there still exists a persistence to align the health needs of military veterans with a medical model. The authors advocate a broader, holistic framework, evident within the suggested care pathway, that acknowledges personal health needs, relationships and social conditions, using a more integrative medical model that combines medical prescribing practices with psychological and psychoeducational practice.

This paper describes the use of holistic, non-medical interventions to improve the mental health and wellbeing of military veterans. A regional project has been identified that will support improved access to both psychological treatments and interventions addressing the wider determinants of mental health. The authors acknowledge the strength of the veteran identity and entrenched social identity assimilated through military service. Through the proposed use of veteran peer-support, the Ty Gwalia project will be coproduced in collaboration with local veteran voluntary organisations, as an empowering lead within the suggested therapeutic framework. Ty Gwalia has the potential to become fully integrated as a pathway for primary care and social prescribing practices, and will strengthen the links between healthcare providers and community, voluntary and statutory services. The project is of importance in seeking to meet what is likely to be an upsurge of mental health issues for military veterans as they deal with the visual reporting of the withdraw from Afghanistan. 

This review acknowledges the role that medication has within a treatment plan, but advocates the use of psychosocial interventions to provide a further opportunity to respond effectively, and at an early stage, to symptoms of mental distress in people living with PTSD, as well as to initiate a more proactive approach to mental health promotion for military veterans. The proposed intervention of horticultural therapy with nutrition advice for military veterans will provide a gateway to community-based resources, improved access to psychological treatments, as well as to services and interventions addressing the wider determinants of mental health.

## Data availability

No data are associated with this article.
