# Supplementary material for: Horticultural therapy, nutrition and post-traumatic stress disorder in post-military veterans: developing non-pharmaceutical interventions to complement existing therapeutic approaches
Source: F1000Res. 2021 Sep 3;10:885. [Version 1] doi: 10.12688/f1000research.70643.1 (PMC8456374; doi:10.12688/f1000research.70643.1)
Supplement: Peer review history

# Peer review: Reviewer response for version 1

**DOI:** 10.5256/f1000research.74245.r93600

**Published:** 2021-09-22

**Authors:** Professor Nicholas Procter

**Affiliations:** 1 Mental Health and Suicide Prevention Research and Education Group, University of South Australia, Adelaide, SA, Australia

It is great to see this paper. What I think is most helpful and beneficial is that as a discursive paper, the descriptive information takes the reader, in a scholarly way, through both the history and thought surrounding non-pharmacological ways of responding to PTSD. The level of detail is good and aligns with the descriptive information provided. The section on neurobiological processes including the interaction between neurotransmitters and lymphocytes is helpful. The work undertaken in nutritional mental health/ psychiatry is progressive in this regard. There is an important body of work helping us to pinpoint and understand the way in which the immune system is involved and implicated in threat detection and threat responses. The information presented is also current- right up to the recent troop withdrawal from Afghanistan.

Is the review written in accessible language?

Yes

Are all factual statements correct and adequately supported by citations?

Yes

Are the conclusions drawn appropriate in the context of the current research literature?

Yes

Is the topic of the review discussed comprehensively in the context of the current literature?

Yes

Reviewer Expertise:

Trauma Responses and Trauma Informed Practice

I confirm that I have read this submission and believe that I have an appropriate level of expertise to confirm that it is of an acceptable scientific standard.

# Peer review: Reviewer response for version 1

**DOI:** 10.5256/f1000research.74245.r94279

**Published:** 2021-09-21

**Authors:** Andrew Williams

**Affiliations:** 1 Institute of Management and Health, University of Wales Trinity Saint David, Carmarthen, UK

An interesting article that is highly topical given the emergence of alternative therapeutic modalities in recent years that is becoming focused under the umbrella term 'social prescribing'. Horticultural therapies are part of an emerging strand of social prescribing known as 'green prescribing' and this paper identifies military veterans with PTSD and continuing/debilitating psychological and mental health concerns as an identifiable study group who can benefit from this alternative approach. The paper is what I would call an 'advocacy paper' recognising the need for research to explore alternative approaches to supporting military veterans with PTSD that can be delivered using horticulture therapy and outlines a future intervention that has been put in place to provide empirical data to inform future 3rd sector and green prescribing commissioning.

Is the review written in accessible language?

Yes

Are all factual statements correct and adequately supported by citations?

Yes

Are the conclusions drawn appropriate in the context of the current research literature?

Yes

Is the topic of the review discussed comprehensively in the context of the current literature?

Yes

Reviewer Expertise:

Social prescribing and ecotherpaies/green therapies linked to physical literacy and social integration. Insider knowledge of military service and the specific issues (including PTSD) facing military veterans.

I confirm that I have read this submission and believe that I have an appropriate level of expertise to confirm that it is of an acceptable scientific standard.

# Peer review: Reviewer response for version 1

**DOI:** 10.5256/f1000research.74245.r93381

**Published:** 2021-09-08

**Authors:** Alun Charles Jones

**Affiliations:** 1 Department of Psychotherapy, Yale Spire Hospital, Wrexham, UK

Thank you for asking me to review this paper. I have read the discussion with care and have the following comments to make:

 This is a generally well-composed paper, which will be of interest to all healthcare practitioners working with trauma, regardless of the origins. The authors are both respected and experienced research academics and have both made long-standing contributions to the research literature and clinical practice for military veterans, experiencing mental and physical ill-health. The paper proposes an ecological or horticultural model of care that is respectful of dignity, non-pharmaceutical, outside of formal psychiatric treatment settings and so is likely to be effective in reducing stigma and shame - both self-to-self and other-to-self.

 It is an approach to care that is also likely to promote a sense of purpose and meaning to participants along with social inclusion, and so facilitate the safe transition from military to civilian life.

 Moreover, the method proposed, also reduces the likelihood of military personnel embarking on a psychiatric career pathway with the disadvantages that this would entail.

 I do, however, think the paper may have a greater impact with the inclusion of a brief discussion of any proposed selection criteria and possible ethical concerns regarding the recruitment of participants - along with some brief thoughts given over to complex PTSD. A paragraph or two would be sufficient and the authors can decide if they consider it appropriate and how best to structure this aspect of their discussion.

 The paper will undoubtedly be followed up by others, at some future date, discussing the benefits gained by participants in the project and, if so, might usefully include those involved with the organisation of treatment and care.

 I wish both authors well with this and future work, which can only provide long-term benefits to the health and well-being of military personnel, their families, and friends.

Is the review written in accessible language?

Yes

Are all factual statements correct and adequately supported by citations?

Yes

Are the conclusions drawn appropriate in the context of the current research literature?

Yes

Is the topic of the review discussed comprehensively in the context of the current literature?

Yes

Reviewer Expertise:

I am a qualified researcher and mental health specialist with experience of working with military personnel experiencing complex PTSD

I confirm that I have read this submission and believe that I have an appropriate level of expertise to confirm that it is of an acceptable scientific standard.
